# Mosquito-borne disease among individuals experiencing homelessness in the USA: a literature review

**DOI:** 10.1007/s10340-026-02042-0

**Published:** 2026-04-22

**Authors:** André B. B. Wilke, Megan D. Hill, Allisandra G. Kummer, Chalmers Vasquez, John-Paul Mutebi, Xi Huo, Han Li, Jing Chen, Shigui Ruan, John C. Beier, Maria Litvinova, Thomas Byrne, Marco Ajelli

**Affiliations:** 1 Department of Epidemiology and Biostatistics, Indiana University School of Public Health, Bloomington, IN USA; 2 Laboratory for Computational Epidemiology and Public Health, Department of Epidemiology and Biostatistics, Indiana University School of Public Health, Bloomington, IN USA; 3 Miami-Dade County Mosquito Control Division, Miami, FL USA; 4 Department of Mathematics, University of Miami, Coral Gables, FL USA; 5 Department of Geography and Sustainable Development, University of Miami, Coral Gables, FL USA; 6 Department of Mathematics, Nova Southeastern University, Fort Lauderdale, FL USA; 7 Department of Public Health Sciences, University of Miami School of Medicine, Miami, FL USA; 8 School of Social Work, Boston College, Boston, MA USA; 9 Center for Healthcare Organization and Implementation Research, VA Bedford Healthcare System, Bedford, MA USA

**Keywords:** *Aedes*, *Anopheles*, *Culex*, Chikungunya, Dengue, Malaria, West Nile, Zika

## Abstract

Mosquito-borne diseases are a major public health issue. The USA has experienced mosquito-borne disease outbreaks, including West Nile virus, Zika, dengue, and malaria, highlighting widespread risk across the country. In the USA, exposure to mosquito vectors primarily occurs outdoors. Individuals experiencing unsheltered homelessness are particularly vulnerable to mosquito-borne diseases due to their increased exposure to mosquito vectors, limited access to healthcare, and high prevalence of underlying conditions. This review aims to examine the literature on mosquito-borne disease infections among individuals experiencing homelessness in the USA between 1999 and 2024. Our search strategy used Boolean methods on PubMed, Scopus, and Web of Science to select relevant publications. Two queries were used: the first query focused on individuals experiencing homelessness and mosquito-borne diseases, and the second one focused on epidemiological analyses of mosquito-borne disease outbreaks in the USA. Only studies reporting at least one infection among individuals experiencing homelessness were included. Our search resulted in the identification of 347 studies. After screening them, eight were ultimately included in this review. Half of the studies identified West Nile virus among individuals experiencing homelessness, and the other half reported cases of malaria. These studies show that individuals who spend more time outdoors, including individuals experiencing homelessness, may be at a greater risk of MBDs compared to the general population. However, the full extent of mosquito-borne disease exposure among individuals experiencing homelessness remains unclear. With climate change and globalization, there is a consensus in the literature showing an increasing risk of mosquito-borne disease transmission in the next decades, and we have already started to witness this increasing trend. Moreover, the USA is experiencing a steep increase in the overall nationwide homeless population. Therefore, we argue that developing policies aimed at providing homeless populations with housing during mosquito-borne disease outbreaks could be instrumental in protecting this segment of the population and promoting health equity.

**Supplementary Information:**

The online version contains supplementary material available at 10.1007/s10340-026-02042-0.

## Introduction

Mosquito-borne diseases (MBDs) represent a constant public health threat. Dengue, Zika, and chikungunya viruses are transmitted primarily by *Aedes aegypti* (Achee et al. 2015). West Nile virus is transmitted by multiple mosquito species, with *Culex quinquefasciatus* and *Culex pipiens* serving as primary vectors in the USA (Centers for Disease Control and Prevention (CDC) 2016). *Anopheles* mosquitoes transmit *Plasmodium* parasites (e.g., *Plasmodium vivax*, *Plasmodium falciparum*), causing malaria in humans (Sinka et al. 2012). Different mosquito species exhibit distinct ecological and physiological traits that shape their vector competence and pathogen transmission.

In the USA, the risk of MBDs varies based on differences in occupational and socioeconomic conditions. In the USA, exposure to mosquito vectors primarily occurs outdoors (Mutebi et al. 2022; Smith et al. 2018) and is predominantly attributed to occupational settings, socioeconomic conditions, and outdoor leisure activities (Martin et al. 2019; Koyoc-Cardeña et al. 2019; Mutebi et al. 2018). For example, blue-collar occupations where the work is conducted primarily outdoors have shown positive associations with the risk of exposure to mosquito vectors (Ventura et al. 2024). Additionally, mosquito vector abundance is higher in low-income communities, increasing the risk of exposure to mosquito vectors in these areas (Martin et al. 2019). This leads to a highly variable health burden and risk of exposure to MBDs for different population groups (Meyer et al. 2007; Molaei et al. 2007; LaDeau et al. 2013; Rothman et al. 2021; Ajelli et al. 2017).

People experiencing unsheltered homelessness—people living in places not meant for human habitation (U.S. 2020)—spend a disproportionate amount of time outdoors (Petrovich and Cronley 2015), increasing their risk of exposure to mosquito vectors. Unsheltered homelessness is a large and growing problem in the USA, and the growth in unsheltered homelessness is responsible for the recent steep increases in the overall nationwide homeless population (U.S. 2020). Currently, approximately 653,000 people are homeless on any given night in the USA, of whom 40% are unsheltered; however, existing data severely underestimate the true size of the unsheltered population (Hopper et al. 2008; U.S. Department of Housing and Urban Development 2023).

Many vulnerable and marginalized populations, who face a higher risk for poor health outcomes, are overrepresented in the unsheltered homeless population. These groups include Black and Indigenous people (Olivet et al. 2021), military veterans, people exiting incarceration, and older adults (Geller and Curtis 2011; Fargo et al. 2012; Culhane et al. 2013). Additionally, individuals experiencing homelessness have worse overall health (Roncarati et al. 2018; Levitt et al. 2009; Montgomery et al. 2016; Nyamathi et al. 2000; Byrne et al. 2016) and are disproportionately affected by other diseases, including mental disorders, substance misuse, and premature death (Fazel et al. 2014). The unsheltered homeless population also has less access to healthcare compared to the housed resident population (Levitt et al. 2009). Consequently, this places them at an increased risk of severe health outcomes in case of an infection (Anderson et al. 2021).

In this study, we conduct a literature review to provide insights into the transmission of mosquito-borne disease infections among the population experiencing homelessness in the USA over the period from 1999 to 2024. To this aim, we consider both studies directly studying MBDs in individuals experiencing homelessness and broader scope studies on autochthonous transmission in the continental USA that reported infections among the homeless population.

## Methods

### Diseases of Interest

We focus on five mosquito-borne diseases: West Nile, dengue, Zika, chikungunya, and malaria. Each of them has a history of local transmission in the USA between 1999 and 2024 (Centers for Disease Control and Prevention (CDC) xxxx). Mosquito vector species responsible for transmitting these pathogens are widespread and abundant (Zardini et al. 2024; Monaghan et al. 2019; Wilke et al. 2023; Levine et al. 2004).

### Search Queries

A search strategy using Boolean methods on PubMed (https://pubmed.ncbi.nlm.nih.gov), Scopus (https://www2.scopus.com), and Web of Science (https://mjl.clarivate.com) was used to select the existing publications about homelessness and mosquito-borne diseases in the USA. The search was conducted on September 17, 2024, considering the title, abstract, and keywords. To conduct this search, we used two independent queries:

- *Query 1* (homeless* OR "transient population" OR "transient communit*") AND (mosquito* OR arbovir* OR dengue OR malaria OR Zika OR chikungunya OR "West Nile" OR DENV OR CHIKV OR ZIKV OR WNV) AND ("United States" OR "USA")
- *Query 2* (Outbreak OR Epidemic) AND (locally acquired OR autochthonous) AND (Mosquito-borne OR Mosquito-Transmitted OR arbovir* OR dengue OR malaria OR Zika OR chikungunya OR "West Nile" OR DENV OR CHIKV OR ZIKV OR WNV) AND ("United States" OR "USA")

For both queries, we limited the search period from 1999, the year when West Nile virus was first introduced in the USA, to 2024. Each query was formatted to function in each searched database.

The first query allows us to identify studies where mosquito-borne diseases in individuals experiencing homelessness in the USA were a main focus of the study (i.e., reported in the title and/or abstract). The second query allows us to retrieve papers whose focus is not on individuals experiencing homelessness, but where cases of mosquito-borne diseases may have been reported in this population group (i.e., individuals experiencing homelessness were reported anywhere in the text). This 2-query strategy allows us to perform a more comprehensive search of the literature relevant for this systematic review.

Each retrieved study was independently assessed by three reviewers (A.B.B.W., M.D.H., and A.G.K.). Any discrepancies in study selection and/or its content were addressed through discussion and consensus.

### Inclusion and Exclusion Criteria

Studies were included if they satisfied the following criteria: (1) the study focused on West Nile, chikungunya, dengue, Zika, or malaria; (2) the study investigated locally acquired infections (i.e., autochthonous) in the USA between 1999 and 2024; (3) the study reported at least one infection among individuals experiencing homelessness. To increase the range of studies included in the analysis, we did not impose any restriction on the diagnostic method.

We excluded study protocols, media news, commentaries, or manuscripts where the full text was unavailable (e.g., conference abstracts).

### Scope of Review and Analysis

In this literature review, we did not perform a meta-analysis, a sensitivity analysis, or other synthesis methods. As such, there is no description of statistical heterogeneity, summary statistics, models, effect measures, or assessment of bias and certainty. Furthermore, no methods were employed to explore causes of heterogeneity, such as subgroup analysis or meta-regression. Therefore, no software packages were utilized or described.

## Results

**Fig. 1:**
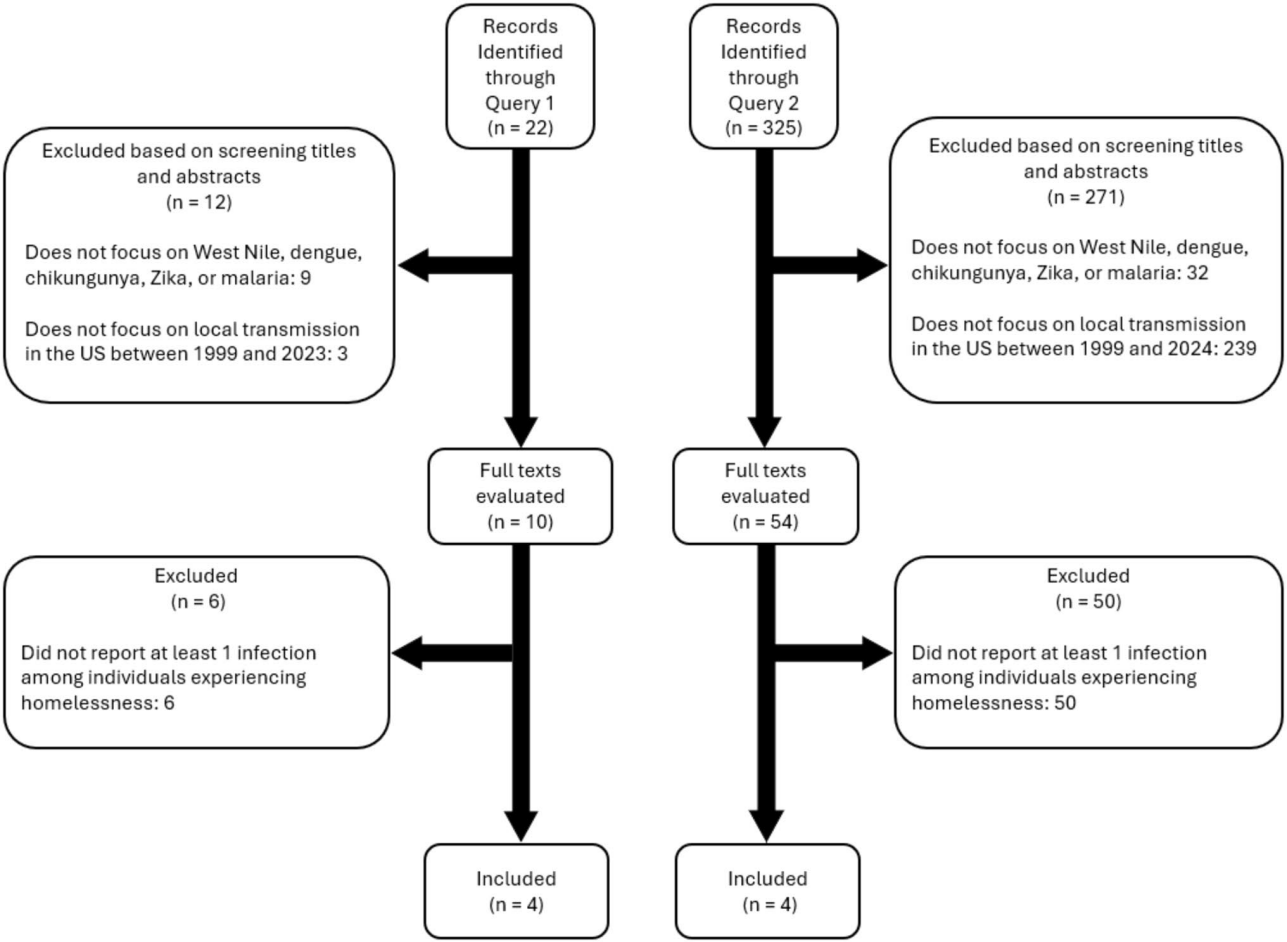
Study flow diagram

Our search resulted in the identification of 347 studies, 22 from Query 1 and 325 from Query 2 (Fig. 1). Among them, 41 studies (9 from Query 1 and 32 from Query 2) were excluded as they did not focus on West Nile, chikungunya, dengue, Zika, or malaria; 242 studies (3 from Query 1 and 239 from Query 2) were excluded as they did not investigate local transmission of one of these diseases in the USA between 1999 and 2024; 56 studies (6 from Query 1 and 50 from Query 2) did not report at least one infection among the population experiencing homelessness. None of the retrieved results was a study protocol, media news, commentary, or a manuscript where the full text was unavailable. As per the inclusion/exclusion criteria, we identified eight articles (4 from Query 1 and 4 from Query 2) to include in this review (Meyer et al. 2007; Murray et al. 2006; Blackburn et al. 2023; Conway et al. 2022; Filler et al. 2006; Eliades et al. 2005; Centers for Disease Control and Prevention (CDC) 2003; Leibler et al. 2016).

The identified studies provide evidence of MBD infection among the homeless population. The two most recent malaria outbreaks in the USA, which took place in 2003 and 2023, reported cases among individuals experiencing homelessness (Blackburn et al. 2023; Eliades et al. 2005; Centers for Disease Control and Prevention (CDC) 2003; Perez et al. 2004). In the 2003 outbreak, one of the eight cases of malaria was an individual experiencing homelessness (Eliades et al. 2005; Perez et al. 2004). The same strain of *Plasmodium vivax* was identified in all eight cases through laboratory hematology (Perez et al. 2004). In 2023, three of the eight locally acquired cases were among individuals experiencing homelessness (Blackburn et al. 2023). Several of the studies identified West Nile virus among individuals experiencing homelessness (Meyer et al. 2007; Murray et al. 2006; Leibler et al. 2016). One study tested for West Nile virus among pets, identifying 10 positive cases where 50% were among pets of unhoused owners (Conway et al. 2022). None of the studies reported evidence of chikungunya virus, dengue virus, or Zika virus in the population experiencing homelessness.

A key element among all the included studies was the emphasis on risk factors associated with MBDs. In particular, they highlight the positive associations between time spent outdoors and the risk of MBDs (Meyer et al. 2007; Filler et al. 2006; Eliades et al. 2005; Centers for Disease Control and Prevention (CDC) 2003), with one study showing that > 6 h of outdoor exposure increases the risk of West Nile virus infection (Meyer et al. 2007). There was also a greater risk of severe health outcomes resulting in hospitalizations among individuals experiencing homelessness compared to the general population (Murray et al. 2006). Furthermore, a positive association was found between the risk of MBDs and HIV infection, as well as risk-taking behaviors (i.e., injection drug use and heavy drinking) among individuals experiencing homelessness (Leibler et al. 2016), supporting the greater risk of severe health outcomes.

Table 1 provides a summary of the findings relevant to the risk of MBDs among individuals experiencing homelessness for all studies identified in this review.

**Table 1:** Summary of studies available in the literature

Reference	Title	Relevant findings for homelessness and MBDs
Murray et al. (2006)	Risk factors for encephalitis and death from West Nile virus infection	Individuals experiencing homelessness showed a higher likelihood of hospitalization due to West Nile compared to the general population
Meyer et al. (2007)	West Nile virus infection among the homeless, Houston, Texas	Factors associated with an increased risk of West Nile infection were found to be chronic homelessness and > 6 h outdoor exposure to mosquitoes
Blackburn et al. (2023)	Outbreak of locally acquired mosquito-transmitted (autochthonous) malaria—Florida and Texas, May–July 2023	Eight cases of locally acquired *Plasmodium vivax* malaria were reported in Florida and Texas. Three of the eight patients were reported experiencing homelessness
Conway et al. (2022)	Benefits, companion animal zoonotic disease prevalence and public perceptions of pet ownership among people experiencing homelessness in northern California	Five out of 42 samples taken from dogs of unhoused owners tested positive for West Nile virus
Eliades et al. (2005)	Malaria surveillance–United States, 2003	In 2003, eight cases of locally transmitted malaria were reported in Palm Beach County, Florida. The 8 affected individuals were found to spend extended periods outdoors, including one homeless individual
CDC et al. (2003)	Local transmission of *Plasmodium vivax* malaria–Palm Beach County, Florida, 2003	Examines seven cases of *Plasmodium vivax* malaria that occurred in Palm Beach County, Florida, during July and August 2003. One case involved a man who had been sleeping in an outdoor homeless camp near a canal
Leibler et al. (2016)	Zoonotic and vector-borne infections among urban homeless and marginalized people in the United States and Europe, 1990–2014	Serological evidence of exposure to pathogens, including *Rickettsia typhi*, West Nile virus, and Seoul hantavirus in individuals experiencing homelessness. HIV infection, injection drug use, and heavy drinking were common risk factors for these infections
Perez et al. (2004)	Hematological laboratory findings in patients of an autochthonous *Plasmodium vivax* malaria outbreak	Hematological findings of the 2003 malaria outbreak in Palm Beach County, Florida, showed that all 8 reported cases, one of which is an individual experiencing homelessness, carried the same strain of *Plasmodium vivax*

## Discussion

Our literature review yielded a small number of studies that examine the transmission of MBD infections among individuals experiencing homelessness in the USA between 1999 and 2024. The eight studies eligible for this review provide evidence of MBD infection among individuals experiencing homelessness. Half of the studies in this review identified West Nile virus among individuals experiencing homelessness. To properly interpret this finding, it is important to consider that among the diseases analyzed in this review, West Nile is the only one endemic in the continental US, with thousands of locally acquired infections reported every year (Centers for Disease Control and Prevention (CDC) xxxx). Our review also provides evidence of malaria cases in the population experiencing homelessness. A possible contributing factor is that both West Nile virus and *Plasmodium* species mosquito vectors are nocturnal, exhibiting heightened host-seeking activity during the night when individuals experiencing homelessness are asleep and thus more exposed to infective bites (Ramin and Svoboda 2009). On the other hand, none of the studies included in this review reported Zika, chikungunya, or dengue among individuals experiencing homelessness. For Zika and chikungunya, this is likely due to the low level of local circulation in the continental USA: 13 reported locally acquired chikungunya virus infections in 2014–2015, and 231 reported locally acquired Zika virus infections in 2016–2017; for both diseases, no other recent locally acquired infections have been reported (Centers for Disease Control and Prevention (CDC) xxxx; Centers for Disease Control and Prevention (CDC) xxxx). Local outbreaks of dengue have occurred each year for the last three years (2022–2024) (Centers for Disease Control and Prevention (CDC) xxxx); however, estimates from the CDC suggest that the number of locally transmitted dengue cases is greatly underreported in the USA and a multiplication factor of 21–105 is needed to correct for the underreporting of the number of laboratory-positive dengue inpatients (i.e., only 1.0–4.8% of infected individuals are confirmed and reported) (Shankar et al. 2018). This highlights the challenges in identifying dengue cases among the population experiencing homelessness.

Our initial search of the literature highlighted the difficulty in identifying literature to include due to the rarity of studies that examine MBDs among individuals experiencing homelessness as their main focus. For this reason, we included a second search query that focused on all local outbreaks in the USA between 1999 and 2024 and included those where at least one infection among the homeless population was reported. However, we expect a high level of underreporting in this population group, given their lower access to healthcare (Roncarati et al. 2018; Levitt et al. 2009; Montgomery et al. 2016; Nyamathi et al. 2000; Byrne et al. 2016). Moreover, the notification rate for the focus diseases is relatively low, and the number of local outbreaks of MBDs in the continental USA is limited.

The identified studies showed that individuals who spend more time outdoors, including individuals experiencing homelessness, may be at a greater risk of MBDs compared to the general population. Identified factors that increase exposure to vector mosquitoes and potentially the risk of MBDs included spending extended periods of time outdoors (Ventura et al. 2024). However, conclusions about the risk factors for MBDs specifically among homeless populations are based on a limited pool of studies and cannot be assessed through a systematic meta-analysis. Nevertheless, these findings suggest important implications about potential risk factors that can be further examined in future studies.

Mosquito control is the most commonly adopted method to reduce MBD risk of transmission and associated health burden (World Health Organization 2012; Lizzi et al. 2014). Other options include installing mosquito screens and AC units (Lindsay et al. 2003; Centers for Disease Control and Prevention (CDC) xxxx). Assistance programs may be set up to provide support to low-income households, or they could be required in rental properties. However, for individuals experiencing homelessness, a group that is greatly expanding nationwide (U.S. 2020), other options should be considered. During the malaria outbreak in Florida in 2003, which included one case among the population experiencing homelessness, the public health response included outreach activities in homeless shelters (Filler et al. 2006). Other targeted strategies may be worth consideration. For example, rapid rehousing pairs time-limited financial assistance used for housing-related expenses with supportive services (e.g., landlord mediation, connections to health services) to move individuals experiencing homelessness into housing as quickly as possible (Byrne et al. 2021). A targeted influx of funding could be used to quickly scale up this intervention to help the unsheltered population get into housing during MBD outbreaks. Another option could be permanent supportive housing, which pairs an ongoing, full housing subsidy with intensive supportive services (e.g., intensive case management, connections to behavioral health services) (Rog et al. 2014) and provides a long-term positive impact on housing stability (Baxter et al. 2019). This intervention could be targeted to certain unsheltered homeless individuals who would otherwise be unable to exit homelessness on their own or with a less intensive intervention like rapid rehousing. Finally, in many communities, congregate emergency shelters (i.e., dormitory/barracks-style arrangements) are the only emergency housing available for people who are homeless, but they cannot accommodate the entire unsheltered population. In response to the COVID-19 pandemic, individuals experiencing homelessness were placed in private hotel and motel rooms (Padgett and Herman 2021), which had positive health, psychological, and housing impacts (Colburn et al. 2020). A similar approach could be used to quickly house a large number of unsheltered persons in the context of an MBD outbreak. Findings reported by Conway et al. (2022) show that dogs of unhoused owners tested positive for West Nile virus, suggesting that zooprophylaxis may reduce disease risk by diverting host-seeking mosquitoes from humans to animals. Even though this mechanism is most relevant for vectors with opportunistic or zoophilic feeding patterns, it has the potential to decrease human–vector contact (Mahande et al. 2007; Brown 1963; Iwashita et al. 2014). The implementation of combinations of these non-mutually exclusive public health interventions targeting both mosquito and human populations could be considered to reduce the risk of mosquito-borne disease transmission.

Given the challenges of implementing the aforementioned policies in the current situation for the USA, which is characterized by a low incidence of MBDs (Centers for Disease Control and Prevention (CDC) xxxx; Centers for Disease Control and Prevention (CDC) xxxx), the effectiveness of these interventions can be tested with mathematical models (Reiner et al. 2013; Smith et al. 2014). These models can be developed to simulate the implementation of interventions in silico prior to their real-world implementation, providing key indications to policymakers as was the case in previous epidemics (Shankar et al. 2018; Lega et al. 2020; Morin et al. 2015; Puggioni et al. 2020; Tjaden et al. 2018). ﻿With climate change and globalization, there is a consensus in the literature showing an increasing risk of MBD transmission in the next decades (Zardini et al. 2024; Monaghan et al. 2019; Gibb et al. 2023; Ryan et al. 2019; Caminade et al. 2014) and policies aimed at reducing the impact of climate change in urban areas (e.g., planting trees, reducing impervious surfaces) could contribute to the proliferation of mosquito vector species (Wilke et al. 2023). We have already started to witness this increasing trend in mosquito-borne disease activity. For example, local dengue outbreaks were reported in the last three years (2022–2024) in Miami-Dade County, Florida (Centers for Disease Control and Prevention (CDC) xxxx), and local transmission of malaria took place in Florida and Texas in 2023 after 20 years of absence (Blackburn et al. 2023; Filler et al. 2006). This highlights the need for strengthening preparedness and response capacity to respond to MBD outbreaks.

In conclusion, we have provided evidence of MBD transmission in the homeless population, but the extent to which individuals experiencing unsheltered homelessness are disproportionately exposed to mosquito-borne diseases remains largely unknown. This vulnerable population already faces significant disparities in mortality rates and various health outcomes (Fazel et al. 2014). Therefore, gaining a better understanding of the scope and impact of their increased vulnerability to mosquito-borne infections is essential for devising policy measures aimed at reducing preventable health inequalities.

## Supplementary Information

Below is the link to the electronic supplementary material.

Supplementary file1 (DOCX 26 KB)

Supplementary file2 (DOCX 31 KB)

## Data Availability

The data supporting this systematic review are drawn from previously reported studies, all of which have been cited. All necessary data for replicating the study’s findings are available within the manuscript.

## References

[CR1] Achee NL, Gould F, Perkins TA, Reiner RC Jr, Morrison AC, Ritchie SA, Gubler DJ, Teyssou R, Scott TW (2015) A critical assessment of vector control for dengue prevention. PLoS Negl Trop Dis 9(5):e0003655 25951103 10.1371/journal.pntd.0003655 PMC4423954

[CR2] Ajelli M, Moise IK, Hutchings TCSG, Brown SC, Kumar N, Johnson NF et al (2017) Host outdoor exposure variability affects the transmission and spread of Zika virus: insights for epidemic control. PLoS Negl Trop Dis 11:e0005851 28910292 10.1371/journal.pntd.0005851 PMC5598931

[CR3] Anderson M-C, Hazel A, Perkins J, Almquist Z (2021) The ecology of unsheltered homelessness: environmental and social-network predictors of well-being among an unsheltered homeless population. Int J Environ Res Public Health 18:7328 34299779 10.3390/ijerph18147328 PMC8306744

[CR4] Baxter AJ, Tweed EJ, Katikireddi SV, Thomson H (2019) Effects of Housing First approaches on health and well-being of adults who are homeless or at risk of homelessness: systematic review and meta-analysis of randomised controlled trials. J Epidemiol Commun Health 73:379–387 10.1136/jech-2018-210981 PMC6581117 30777888

[CR5] Blackburn D, Drennon M, Broussard K, Morrison AM, Stanek D, Sarney E et al (2023) Outbreak of locally acquired mosquito-transmitted (autochthonous) malaria - Florida and Texas, May-July 2023. MMWR Morb Mortal Wkly Rep 72:973–978 37676839 10.15585/mmwr.mm7236a1 PMC10495185

[CR6] Brown HW (1963) Practical malariology. JAMA, J Am Med Assoc 186:87

[CR7] Byrne T, Montgomery AE, Fargo JD (2016) Unsheltered homelessness among veterans: correlates and profiles. Commun Ment Health J 52:148–157 10.1007/s10597-015-9922-0 26289119

[CR8] Byrne T, Huang M, Nelson RE, Tsai J (2021) Rapid rehousing for persons experiencing homelessness: a systematic review of the evidence. Hous Stud. 10.1080/02673037.2021.1900547

[CR9] Caminade C, Kovats S, Rocklov J, Tompkins AM, Morse AP, Colón-González FJ et al (2014) Impact of climate change on global malaria distribution. Proc Natl Acad Sci 111:3286–3291 24596427 10.1073/pnas.1302089111 PMC3948226

[CR10] Centers for Disease Control and Prevention (CDC) (2003) Local transmission of *Plasmodium vivax* malaria--Palm Beach County, Florida, 2003. MMWR Morb Mortal Wkly Rep 52:908–911 14508439

[CR11] Centers for Disease Control and Prevention (CDC). West Nile virus disease cases reported to CDC by state of residence. Available at: https://www.cdc.gov/west-nile-virus/data-maps/index.html

[CR12] Centers for Disease Control and Prevention (CDC). Chikungunya disease cases reported to CDC by state of residence. Available at: https://www.cdc.gov/chikungunya/data-maps/chikungunya-us.html.

[CR13] Centers for Disease Control and Prevention (CDC). Arbovirus Catalog. Available at: https://wwwn.cdc.gov/arbocat/

[CR14] Centers for Disease Control and Prevention (CDC). Dengue disease cases reported to CDC by state of residence. Available at: https://www.cdc.gov/dengue/areaswithrisk/in-the-us.html.

[CR15] Centers for Disease Control and Prevention (CDC). Mosquito species in which West Nile virus has been detected. 2016. Available at: https://www.cdc.gov/westnile/resources/pdfs/MosquitoSpecies1999-2012.pdf

[CR16] Centers for Disease Control and Prevention, Filler SJ, MacArthur JR, Parise M, Wirtz R, Eliades MJ et al (2006) Locally acquired mosquito-transmitted malaria: a guide for investigations in the United States. MMWR Recomm Rep 55:1–9 16960552

[CR17] Centers for Disease Control and Prevention (CDC). Zika disease cases reported to CDC by state of residence. Available at: https://www.cdc.gov/zika/zika-cases-us/index.html.

[CR18] Centers for Disease Control and Prevention (CDC). West Nile Virus in the United States: Guidelines for Surveillance, Prevention. Available at: https://www.cdc.gov/westnile/resources/pdfs/wnvGuidelines.pdf

[CR19] Colburn G, Fyall R, Thompson S, et al. (2020) Impact of hotels as non-congregate emergency shelters: An analysis of investments in hotels as emergency shelter in King County, WA during the COVID-19 pandemic. University of Washington. Available at: https://kcrha.org/wp-content/uploads/2020/11/Impact-of-Hotels-as-ES-Study_Full-Report_Final-11302020.pdf 10.1080/10511482.2022.2075027 PMC10586465 37860162

[CR20] Conway KL, Jasuja RM, Hauser NE, Foley JE (2022) Benefits, companion animal zoonotic disease prevalence and public perceptions of pet ownership among people experiencing homelessness in Northern California. Zoonoses Public Health 69:806–815 35603643 10.1111/zph.12970

[CR21] Culhane DP, Metraux S, Byrne T, Stino M, Bainbridge J (2013) The age structure of contemporary homelessness: evidence and implications for public policy. Anal Soc Issues Public Policy 13:228–244

[CR22] Eliades MJ, Shah S, Nguyen-Dinh P, Newman RD, Barber AM, Nguyen-Dinh P et al (2005) Malaria surveillance–United States, 2003. MMWR Surveill Summ 54:25–40 15931154

[CR23] Fargo J, Metraux S, Byrne T, Munley E, Montgomery AE, Jones H (2012) Prevalence and risk of homelessness among US veterans. Prev Chronic Dis 9:E45 22280960 PMC3337850

[CR24] Fazel S, Geddes JR, Kushel M (2014) The health of homeless people in high-income countries: descriptive epidemiology, health consequences, and clinical and policy recommendations. Lancet 384:1529–1540 25390578 10.1016/S0140-6736(14)61132-6 PMC4520328

[CR25] Geller A, Curtis MA (2011) A sort of homecoming: incarceration and the housing security of urban men. Soc Sci Res 40:1196–1213 21927519 10.1016/j.ssresearch.2011.03.008 PMC3173782

[CR26] Gibb R, Colón-González FJ, Lan PT, Huong PT, Nam VS, Duoc VT et al (2023) Interactions between climate change, urban infrastructure and mobility are driving dengue emergence in Vietnam. Nat Commun 14:8179 38081831 10.1038/s41467-023-43954-0 PMC10713571

[CR27] Hopper K, Shinn M, Laska E, Meisner M, Wanderling J (2008) Estimating numbers of unsheltered homeless people through plant-capture and postcount survey methods. Am J Public Health 98:1438–1442 17901451 10.2105/AJPH.2005.083600 PMC2446453

[CR28] Iwashita H, Dida GO, Sonye GO, Sunahara T, Futami K, Njenga SM, Chaves LF, Minakawa N (2014) Push by a net, pull by a cow: can zooprophylaxis enhance the impact of insecticide treated bed nets on malaria control? Parasit Vectors 7:52 24472517 10.1186/1756-3305-7-52 PMC3917899

[CR29] Koyoc-Cardeña E, Medina-Barreiro A, Cohuo-Rodríguez A, Pavía-Ruz N, Lenhart A, Ayora-Talavera G et al (2019) Estimating absolute indoor density of *Aedes aegypti* using removal sampling. Parasit Vectors 12:250 31113454 10.1186/s13071-019-3503-y PMC6528352

[CR30] LaDeau S, Leisnham P, Biehler D, Bodner D (2013) Higher mosquito production in low-income neighborhoods of Baltimore and Washington, DC: understanding ecological drivers and mosquito-borne disease risk in temperate cities. Int J Environ Res Public Health 10:1505–1526 23583963 10.3390/ijerph10041505 PMC3709331

[CR31] Lega J, Brown HE, Barrera R (2020) A 70% Reduction in mosquito populations does not require removal of 70% of mosquitoes. J Med Entomol 57:1668–1670 32300803 10.1093/jme/tjaa066 PMC7566742

[CR32] Leibler JH, Zakhour CM, Gadhoke P, Gaeta JM (2016) Zoonotic and vector-borne infections among urban homeless and marginalized people in the United States and Europe, 1990–2014. Vector Borne Zoonotic Dis 16:435–444 27159039 10.1089/vbz.2015.1863

[CR33] Levine RS, Peterson AT, Benedict MQ (2004) Distribution of members of Anopheles quadrimaculatus say s.l. (Diptera: Culicidae) and implications for their roles in malaria transmission in the United States. J Med Entomol 41:607–613 15311451 10.1603/0022-2585-41.4.607

[CR34] Levitt AJ, Culhane DP, DeGenova J, O’Quinn P, Bainbridge J (2009) Health and social characteristics of homeless adults in Manhattan who were chronically or not chronically unsheltered. Psychiatr Serv 60:978–981 19564231 10.1176/ps.2009.60.7.978

[CR35] Lindsay SW, Jawara M, Paine K, Pinder M, Walraven GEL, Emerson PM (2003) Changes in house design reduce exposure to malaria mosquitoes. Trop Med Int Health 8:512–517 12791056 10.1046/j.1365-3156.2003.01059.x

[CR36] Lizzi KM, Qualls WA, Brown SC, Beier JC (2014) Expanding Integrated Vector Management to promote healthy environments. Trends Parasitol 30:394–400 25028090 10.1016/j.pt.2014.06.001 PMC4112142

[CR37] Mahande A, Mosha F, Mahande J, Kweka E (2007) Feeding and resting behaviour of malaria vector, *Anopheles arabiensis* with reference to zooprophylaxis. Malar J 6:100 17663787 10.1186/1475-2875-6-100 PMC1964787

[CR38] Martin E, Medeiros MCI, Carbajal E, Valdez E, Juarez JG, Garcia-Luna S et al (2019) Surveillance of *Aedes aegypti* indoors and outdoors using Autocidal Gravid Ovitraps in South Texas during local transmission of Zika virus, 2016 to 2018. Acta Trop 192:129–137 30763563 10.1016/j.actatropica.2019.02.006

[CR39] Meyer TE, Bull LM, Holmes KC, Pascua RF, Travassos da Rosa A, Gutierrez CR et al (2007) West Nile virus infection among the Homeless, Houston, Texas. Emerg Infect Dis 13:1500–1503 18257995 10.3201/eid1310.070442 PMC2851536

[CR40] Molaei G, Andreadis TG, Armstrong PM, Bueno R, Dennett JA, Real SV et al (2007) Host feeding pattern of *Culex quinquefasciatus* (Diptera: Culicidae) and its role in transmission of West Nile virus in Harris County, Texas. Am J Trop Med Hyg 77:73–81 17620633

[CR41] Monaghan AJ, Eisen RJ, Eisen L, McAllister J, Savage HM, Mutebi JP et al (2019) Consensus and uncertainty in the geographic range of *Aedes aegypti* and *Aedes albopictus* in the contiguous United States: multi-model assessment and synthesis. PLoS Comput Biol 15:1–19 10.1371/journal.pcbi.1007369 PMC6786520 31600194

[CR42] Montgomery AE, Szymkowiak D, Marcus J, Howard P, Culhane DP (2016) Homelessness, unsheltered status, and risk factors for mortality. Public Health Rep 131:765–772 28123222 10.1177/0033354916667501 PMC5230839

[CR43] Morin CW, Monaghan AJ, Hayden MH, Barrera R, Ernst K (2015) Meteorologically driven simulations of dengue epidemics in San Juan. PR Plos Negl Trop Dis 9:e0004002 26275146 10.1371/journal.pntd.0004002 PMC4537107

[CR44] Murray KO, Baraniuk S, Resnick M, Arafat R, Kilborn C, Cain K et al (2006) Risk factors for encephalitis and death from West Nile virus infection. Epidemiol Infect 134:1325–1332 16672108 10.1017/S0950268806006339 PMC2870518

[CR45] Mutebi JP, Hughes HR, Burkhalter KL, Kothera L, Vasquez C, Kenney JL (2018) Zika virus MB16-23 in mosquitoes, Miami-Dade County, Florida, USA, 2016. Emerg Infect Dis 24:808–810 29400646 10.3201/eid2404.171919 PMC5875261

[CR46] Mutebi J-P, Wilke ABB, Ostrum E, Vasquez C, Cardenas G, Carvajal A et al (2022) Diel activity patterns of two distinct populations of *Aedes aegypti* in Miami, FL and Brownsville. TX Sci Rep 12:5315 35351905 10.1038/s41598-022-06586-w PMC8964714

[CR47] Nyamathi AM, Leake B, Gelberg L (2000) Sheltered versus nonsheltered homeless women. J Gen Intern Med 15:565–572 10940149 10.1046/j.1525-1497.2000.07007.x PMC1495574

[CR48] Olivet J, Wilkey C, Richard M, Dones M, Tripp J, Beit-Arie M et al (2021) Racial inequity and homelessness: findings from the SPARC Study. Ann Am Acad Pol Soc Sci 693:82–100

[CR49] Padgett DK, Herman D (2021) From shelters to hotels: an enduring solution to ending homelessness for thousands of Americans. Psychiatr Serv 72:986–987 34253037 10.1176/appi.ps.202100170

[CR50] Perez MT, Morand J, Bush LM, Crankshaw K, Sudduth NC (2004) Hematological laboratory findings in patients of an autochthonous *Plasmodium vivax* malaria outbreak. Lab Med 35:420–426

[CR51] Petrovich JC, Cronley CC (2015) Deep in the heart of Texas: a phenomenological exploration of unsheltered homelessness. Am J Orthopsychiatry 85:315–323 25602352 10.1037/ort0000043

[CR52] Puggioni G, Couret J, Serman E, Akanda AS, Ginsberg HS (2020) Spatiotemporal modeling of dengue fever risk in Puerto Rico. Spat Spatiotemporal Epidemiol 35:100375 33138945 10.1016/j.sste.2020.100375

[CR53] Ramin B, Svoboda T (2009) Health of the homeless and climate change. J Urban Health 86:654–664 19444615 10.1007/s11524-009-9354-7 PMC2704276

[CR54] Reiner RC, Perkins TA, Barker CM, Niu T, Chaves LF, Ellis AM et al (2013) A systematic review of mathematical models of mosquito-borne pathogen transmission: 1970–2010. J R Soc Interface 10:20120921 23407571 10.1098/rsif.2012.0921 PMC3627099

[CR55] Rog DJ, Marshall T, Dougherty RH, George P, Daniels AS, Ghose SS et al (2014) Permanent supportive housing: assessing the evidence. Psychiatr Serv 65:287–294 24343350 10.1176/appi.ps.201300261

[CR56] Roncarati JS, Baggett TP, O’Connell JJ, Hwang SW, Cook EF, Krieger N et al (2018) Mortality among unsheltered homeless adults in Boston, Massachusetts, 2000-2009. JAMA Intern Med 178:1242 30073282 10.1001/jamainternmed.2018.2924 PMC6142967

[CR57] Rothman SE, Jones JA, Ladeau SL, Leisnham PT (2021) Higher West Nile virus infection in *Aedes albopictus* (Diptera: Culicidae) and *Culex* (Diptera: Culicidae) mosquitoes from lower income neighborhoods in urban Baltimore, MD. J Med Entomol 58:1424–1428 33257956 10.1093/jme/tjaa262

[CR58] Ryan SJ, Carlson CJ, Mordecai EA, Johnson LR (2019) Global expansion and redistribution of *Aedes*-borne virus transmission risk with climate change. PLoS Negl Trop Dis 13:e0007213 30921321 10.1371/journal.pntd.0007213 PMC6438455

[CR59] Shankar MB, Rodríguez-Acosta RL, Sharp TM, Tomashek KM, Margolis HS, Meltzer MI (2018) Estimating dengue under-reporting in Puerto Rico using a multiplier model. PLoS Negl Trop Dis 12:e0006650 30080848 10.1371/journal.pntd.0006650 PMC6095627

[CR60] Sinka ME, Bangs MJ, Manguin S, Rubio-Palis Y, Chareonviriyaphap T, Coetzee M, Mbogo CM, Hemingway J, Patil AP, Temperley WH, Gething PW, Kabaria CW, Burkot TR, Harbach RE, Hay SI (2012) A global map of dominant malaria vectors. Parasit Vectors 5:69 22475528 10.1186/1756-3305-5-69 PMC3349467

[CR61] Smith DL, Perkins TA, Reiner RC, Barker CM, Niu T, Chaves LF et al (2014) Recasting the theory of mosquito-borne pathogen transmission dynamics and control. Trans R Soc Trop Med Hyg 108:185–197 24591453 10.1093/trstmh/tru026 PMC3952634

[CR62] Smith M, Dixon D, Bibbs C, Autry D, Xue RD (2018) Diel patterns of *Aedes aegypti* (Diptera: Culicidae) after resurgence in St. Augustine, Florida as collected by a mechanical rotator trap. J Vector Ecol 43:201–204 29757509 10.1111/jvec.12302

[CR63] Tjaden NB, Caminade C, Beierkuhnlein C, Thomas SM (2018) Mosquito-borne Diseases: advances in modelling climate-change impacts. Trends Parasitol 34:227–245 29229233 10.1016/j.pt.2017.11.006

[CR64] U.S. Department of Housing and Urban Development (2021) The 2020 Annual Homeless Assessment Report to Congress, Part 1: Point-In-Time Estimates of Homelessness. Washington, DC

[CR65] U.S. Department of Housing and Urban Development (2023) The Annual Homeless Assessment Report to Congress, Part 1: Point-in-Time Estimates of Homelessness. U.S. Department of Housing and Urban Development, Washington, DC

[CR66] Ventura PC, Wilke ABB, Chitturi J, Kummer AG, Agrawal S, Vasquez C, et al. (2024) Unveiling the role of mosquito and human diel activity patterns in the risk of mosquito-borne disease infection. medRxiv; 10.1101/2024.05.29.24308139

[CR67] Wilke ABB, Vasquez C, Beier JC (2023) Presence and abundance of malaria vector species in Miami-Dade County, Florida. Malar J. 10.1186/s12936-024-04847-9 10.1186/s12936-024-04847-9 PMC10797977 38238772

[CR68] Wilke ABB, Chang NB, Townsend J, Benelli G, Ajelli M (2023) Unintended effects of urban policies on the risk of arbovirus transmission. Trends Parasitol 39:1001–1003 37739907 10.1016/j.pt.2023.08.012

[CR69] World Health Organization (2012) Handbook for integrated vector management. World Health Organization, Geneva

[CR70] Zardini A, Menegale F, Gobbi A, Manica M, Guzzetta G, D’Andrea V et al (2024) Estimating the potential risk of transmission of arboviruses in the Americas and Europe: a modelling study. Lancet Planet Health 8:e30–e40 38199719 10.1016/S2542-5196(23)00252-8

